# Complex furrows in a 2D epithelial sheet code the 3D structure of a beetle horn

**DOI:** 10.1038/s41598-017-14170-w

**Published:** 2017-10-24

**Authors:** Keisuke Matsuda, Hiroki Gotoh, Yuki Tajika, Takamichi Sushida, Hitoshi Aonuma, Teruyuki Niimi, Masakazu Akiyama, Yasuhiro Inoue, Shigeru Kondo

**Affiliations:** 1 Graduate School of Frontier Bioscience, Osaka University, Suita, Osaka 565-0871 Japan; 2 Graduate School of Bioagricultural Sciences, Nagoya University, Nagoya, Aichi 464-8601 Japan; 3 Graduate School of Medicine, Gunma University, Maebashi, Gunma 371-8511 Japan; 4 Research Institute for Electronic Science, Hokkaido University, Sapporo, Hokkaido 060-0812 Japan; 5 Division of Evolutionary Developmental Biology, National institute for basic biology, Okazaki, Aichi 444-8585 Japan; 6 Institute for Frontier Life and Medical Sciences, Kyoto University, Kyoto, Kyoto, 606-8507 Japan

## Abstract

The external organs of holometabolous insects are generated through two consecutive processes: the development of imaginal primordia and their subsequent transformation into the adult structures. During the latter process, many different phenomena at the cellular level (e.g. cell shape changes, cell migration, folding and unfolding of epithelial sheets) contribute to the drastic changes observed in size and shape. Because of this complexity, the logic behind the formation of the 3D structure of adult external organs remains largely unknown. In this report, we investigated the metamorphosis of the horn in the Japanese rhinoceros beetle *Trypoxylus dichotomus*. The horn primordia is essentially a 2D epithelial cell sheet with dense furrows. We experimentally unfolded these furrows using three different methods and found that the furrow pattern solely determines the 3D horn structure, indicating that horn formation in beetles occurs by two distinct processes: formation of the furrows and subsequently unfolding them. We postulate that this developmental simplicity offers an inherent advantage to understanding the principles that guide 3D morphogenesis in insects.

## Introduction

Elucidating the mechanisms that generate the characteristic 3D body shape of an animal is one of the major issues in the biological sciences. During the past three decades, our understanding of embryonic development has deepened remarkably. The mechanisms that guide morphogenetic processes at later stages, however, remain largely unknown.

The body surface of arthropods is covered by an exoskeleton, composed of hard cuticle with limited elasticity^1^. As this exoskeleton is located at the outermost region of the animal, it cannot be enlarged during growth^1,2^. Consequently, many arthropod species employ a different strategy to enable growth: molting, during which the old exoskeleton is replaced by new cuticle. As the new exoskeleton is formed at the inner side of the older one, it is initially somewhat “smaller”. However, the newly formed cuticle contains furrows which are extended during molting, eventually allowing a bigger body size^1–3^. During each consecutive molt, the animal gets bigger while its outer shape can become more complex. Nevertheless, as the old exoskeleton serves as the structural template upon which the epithelial sheet secretes the new cuticle, such 3D shape changes are in most cases not very drastic. However, during metamorphosis, entirely new cuticular structures can appear on the exoskeleton of adult holometabolous insects as the result of the unfolding of imaginal primordia, epithelial sheets kept aside during larval and pupal stages and undergoing a separate developmental program.

Development of imaginal primordia (or imaginal discs) has been well studied in *Drosophila* ^4–9^. At the first instar larval stage, each imaginal disc initially develops as a small sac of epithelial cells, which grows and develops a series of concentric furrows during subsequent larval and prepupal stages. During the pupal stage, these furrows are extended and as such the imaginal disc everts and elongates, thereby transforming itself into a wing or leg^10,11^. During metamorphosis, many other cellular phenomena – such as proliferation, migration, cell shape changes and convergent extension – contribute to the final 3D shape of the adult appendage^4,12–14^. At present, this complexity significantly hampers the elucidation of the mechanisms involved, preventing a thorough understanding of the principles that guide 3D morphogenesis in insects.

Conversely, studying morphogenetic mechanisms in a species whereby massive body transformations exclusively occur as the result of epithelial sheet unfolding should be easier. Indeed, such drastic shape changes can be simulated by computer algorithms, allowing predictions of a 3D shape based solely upon information concerning the initial furrow pattern.

The horn primordia of horned beetles have extensively complex furrows and are compactly packed in the larval head^15–18^. During pupation, the horn primordia elongates extensively to generate the pupal horn (which in many species has nearly the same size as the adult beetle horn). In the Japanese rhinoceros beetle *Trypoxylus dichotomus*, pupation is completed within two hours, suggesting that the contribution of other cellular activities is absent or very limited. In this report, we demonstrate that the furrow pattern of the beetle horn primordia possesses all the necessary information to instruct the formation of the pupal horn. Our results therefore suggest that the beetle horn is a useful experimental system that can facilitate a comprehensive understanding of 3D morphogenesis.

## Results and Discussion

### Anatomy of the horn primordia of *Trypoxylus dichotomus*

**Figure 1:**
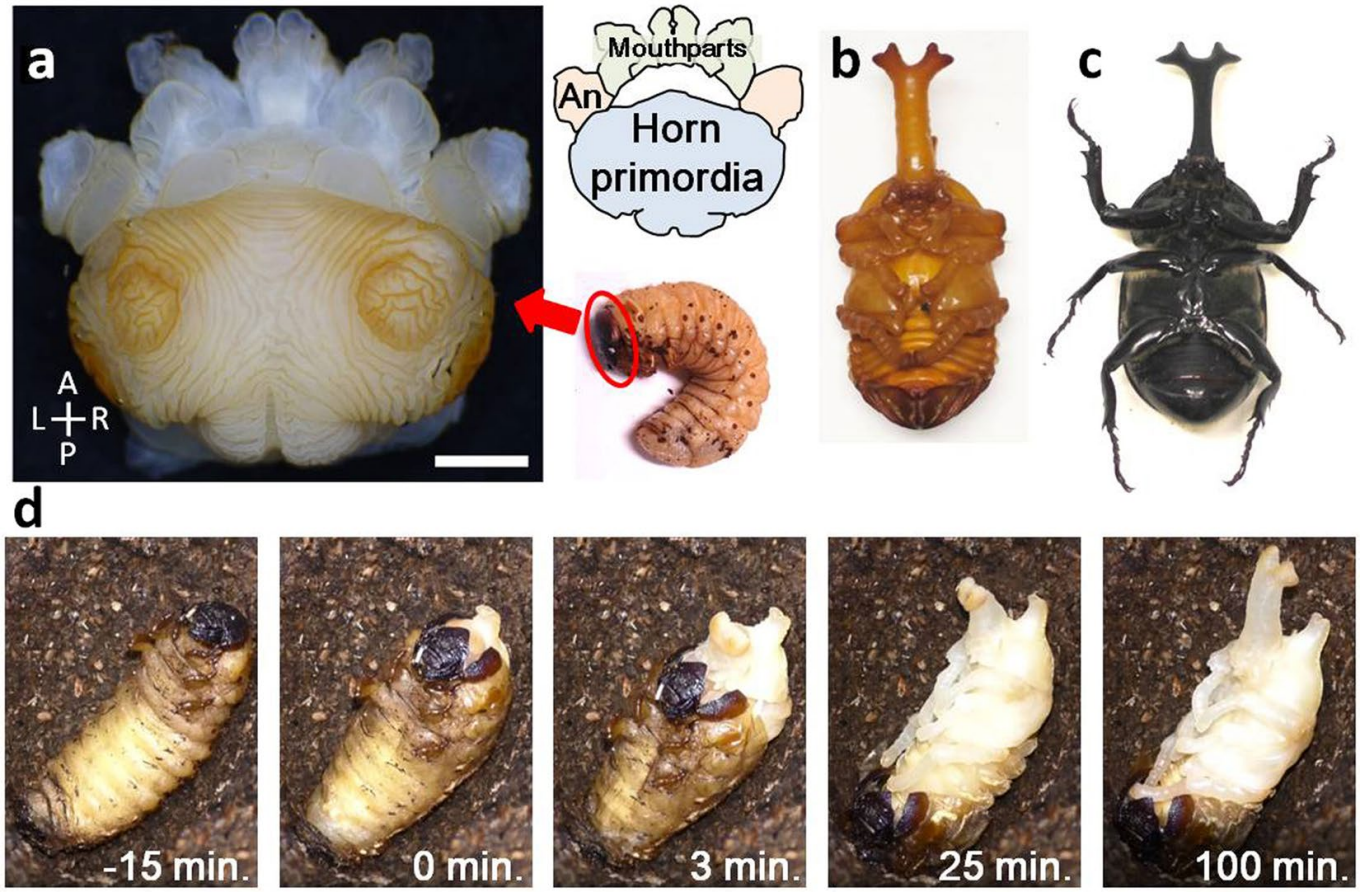
Pupation and horn primordia of the Japanese rhinoceros beetle *Trypoxylus dichotomus*. (**a**) Horn primordia of *Trypoxylus dichotomus*. Larval cuticle was removed. (**b**) Ventral view of a male *Trypoxylus dichotomus* pupa. The large horn appears at this stage. (**c**) Ventral view of a male *Trypoxylus dichotomus* adult. Note that both length and shape of the adult horn remain basically unchanged from its pupal form. (**d**) Time lapse images of pupal molt. Larval cuticle was removed within 30 min after initiation of the molting process. Unfolding of the horn primordia by internal haemolymph pressure was mostly completed within two hours.

**Figure 2:**
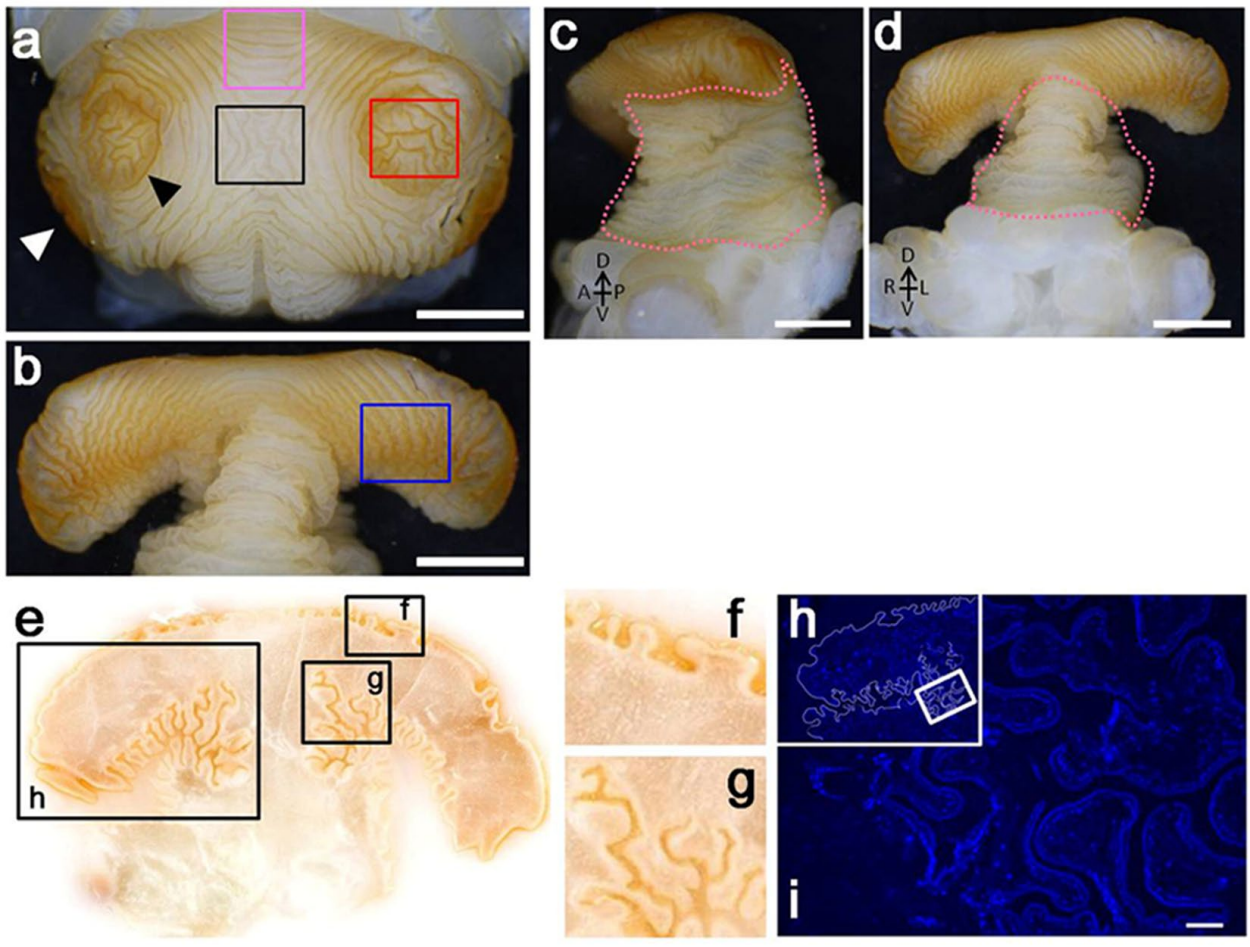
Complex furrows on the surface of the horn primordial. (**a** to **d**) Photos of a completely formed horn primordia dissected from larval (prepupal) head. (white scale bars indicate 2 mm) (**a**) Top of the cap region. Two concentric circles can be clearly recognized. (**b**) Underside of the cap region. (**c** and **d**) Side view and frontal view of the horn primordia, respectively. Lifting the cap of the horn primordia exposes the accordion-like folding pattern of the stalk (pink dotted lines). (**e**) Cryostat frontal section of a fully developed horn primordia: depth and pattern of the furrows differ among different regions. (**f**) Furrows at the top of the cap region are less deep, compared to furrows in other regions. (**g**) Many deep and branched furrows can be recognized at the underside of the cap. (**h**) Hoechst stained section of a horn primordia (region corresponds to window in (**e**). The border of the horn primordia is artificially outlined as a white line. (**i**) Magnified image of the region depicted by a window in (**h**). Cells are aligned along the surface of the furrows. No solid tissue is present inside the primordia (scale bar indicates 100 µm).

The horn imaginal primordia of the Japanese rhinoceros beetle *Trypoxylus dichotomus* develops beneath the head capsule of the larva, during the prepupal stage (Fig. 1a, Supplementary Movie S1). During pupation, the horn primordia rapidly (~ two hours) unfolds to form the long pupal horn (Fig. 1b–d). As the shape of the pupal horn primordia resembles a mushroom, we named its top broad region the “cap” (Fig. 2a,b) and the vertical column region the “stalk” (Fig. 2c,d). The surface of the horn primordia shows a characteristic furrow pattern, whereby the spacing between each furrow, the directionality and the curvature of the furrows differ among different regions of the horn primordia. On the surface of the cap, a pair of distinguished concentric furrows can be observed (Fig. 2a, black arrowhead), whereas at the edge of the cap, two additional, less distinct concentric furrows are present (Fig. 2a, white arrowhead). The furrows in the center of these concentric circles look randomly arranged. These positions correspond to the four tips of the pupal horn.

By lifting the cap with a forceps, the furrows on the underside of both cap and stalk can be observed (Fig. 2b). The furrows along the underside of the cap run primarily in an anteroposterior direction (Fig. 2b, blue window). These furrows are mostly straight (Fig. 2a, pink window), but in some regions they run along a zigzag pattern (Fig. 2b, blue window). Furrows on the stalk region run in the direction circling the stem, thereby mimicking an accordion-like structure. A frontal section of the horn primordia shows that the depth of the furrows differs among the different regions of the horn primordia (Fig. 2c). Furrows at the top of the cap (Fig. 2f) are less deep than folds at the underside of the cap. Furrows at the stalk (Fig. 2g) are more complex, as they are very deep and extensively bifurcated. As the horn primordia are composed of a folded single layer of cells (Fig. 1h,i) filled with haemolymph (an amorphous body fluid), it is only reasonable to assume that the information to build the horn should be imprinted exclusively in this folded cell sheet. Or in other words: it is expected that the furrow pattern of the horn primordia will determine the ultimate 3D shape of the adult horn.

### Cell division and migration are not involved in extension of the horn primordia

To test if the furrow pattern of the horn primordia will determine the ultimate 3D shape of the adult horn, we performed two simple experiments. Although unfolding of the horn primordia occurs within a few hours, some cellular events (e.g. cell shape changes, cell division, sorting or migration) could contribute to the formation of the 3D structure of the horn. To reduce the effects of such cellular events, we experimentally shortened the period of horn primordia transformation.

**Figure 3:**
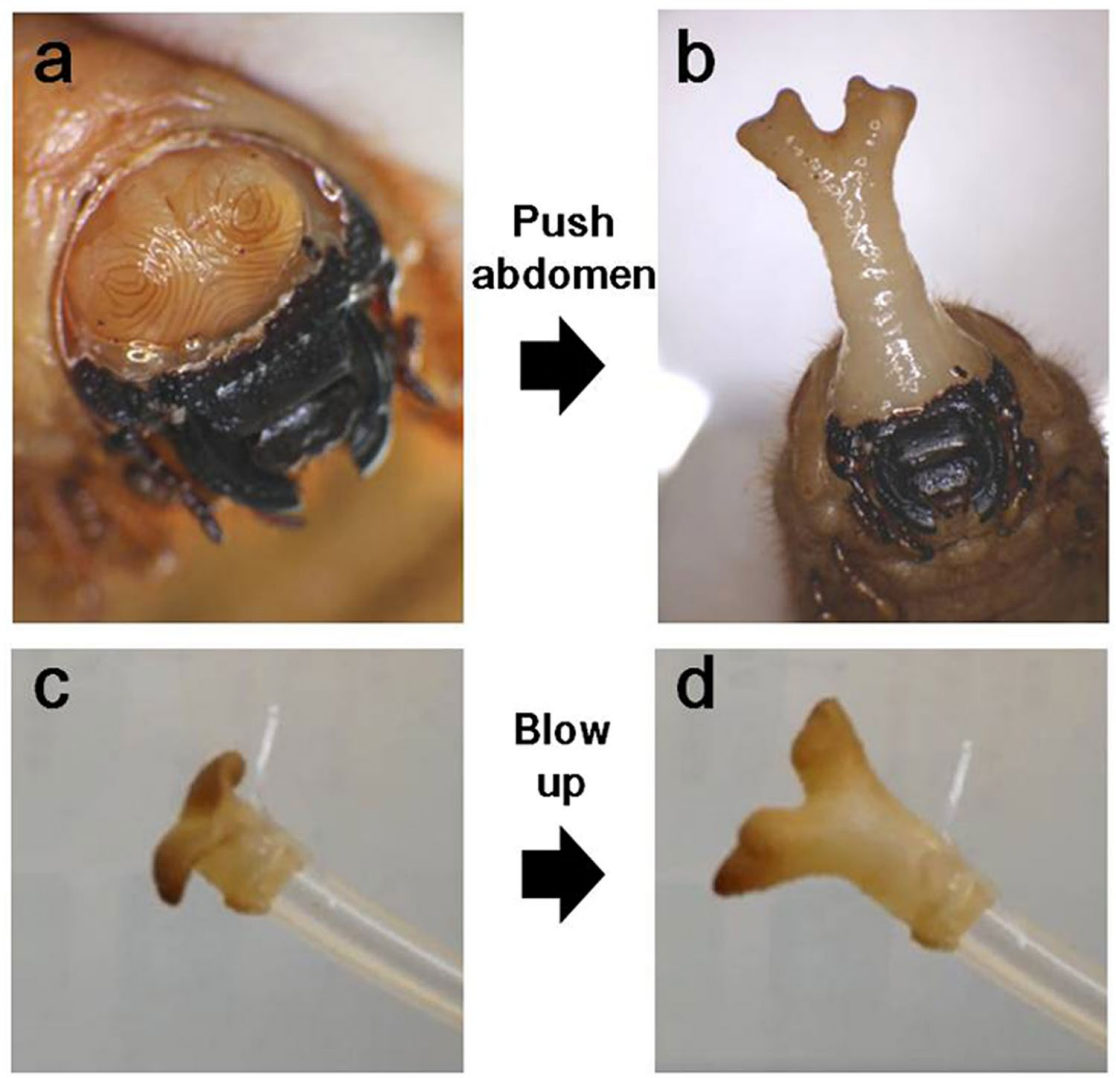
Physical extension of the horn primordial. (**a**,**b**) Unfolding of an intact horn primordia. (**a**) The prepupal horn primordia was exposed by artificially removing the head cuticle. (**b**) The horn primordia was unfolded (within one minute) as a result of pushing the prepupal abdomen, thereby increasing heamolymph pressure. The bifurcated 3D shape of the thus unfolded horn is very similar to that of the natural pupal horn. (**c**,**d**) Unfolding of a chemically fixed horn primordia. (**c**) The dissected horn primordia was fixed with formalin and connected to a plastic tube. (**d**) The horn primordia was blown up (within seconds) as a result of air pressure unfolding the furrows. Its extended shape and size strongly resemble the natural pupal horn.

Just before pupation, we removed the head capsule of the larva with a forceps as to expose the horn primordia (Fig. 3a). Then, we pushed the larval abdomen in order to pump the haemolymph into the anterior part of the body. As a result of the mechanical pressure thus employed, the horn primordia is forced to unfold (within less than 1 minute), thereby giving rise to a fully extended horn without allowing sufficient time for cellular movement, active cell shape changes, or other cellular mechanisms to contribute to the final proportions of the horn. Horn rapidly inflated in this fashion had the correct 3D shape of a naturally-extended pupal horn (Fig. 3b). In a second experiment, the horn primordia was fixed with formalin, whereupon air was blown into the primordia through a silicon tube connected to the base of the stalk. Following air pressure thus employed, the horn primordia correctly unfolded (within seconds) to form a structure with the appropriate 3D shape of a natural horn. As the tissue was chemically fixed with a cross-linking agent, primordia unfurling can apparently proceed in the absence of any living cells (Fig. 3c,d, Supplementary Movie S2). Taken together, these experiments strongly suggest that patterns of epidermal furrowing are sufficient to account for full transformation of the horn primordia into a pupal horn.

### Computer simulation of horn imaginal primordia unfolding

The above experiments suggest that unfolding of the horn primordia furrows is the major determinant of the 3D beetle horn shape. However, since cuticle layers can be somewhat elastic, local extension or compression of the cuticle sheet could also be involved in 3D shape formation. To evaluate this possibility and its contribution on primordia-final horn transformation, we recorded the 3D shape of the horn primordia as to construct a computer model, allowing us to simulate the 3D shape changes that accompany its transformation into a pupal horn.

**Figure 4:**
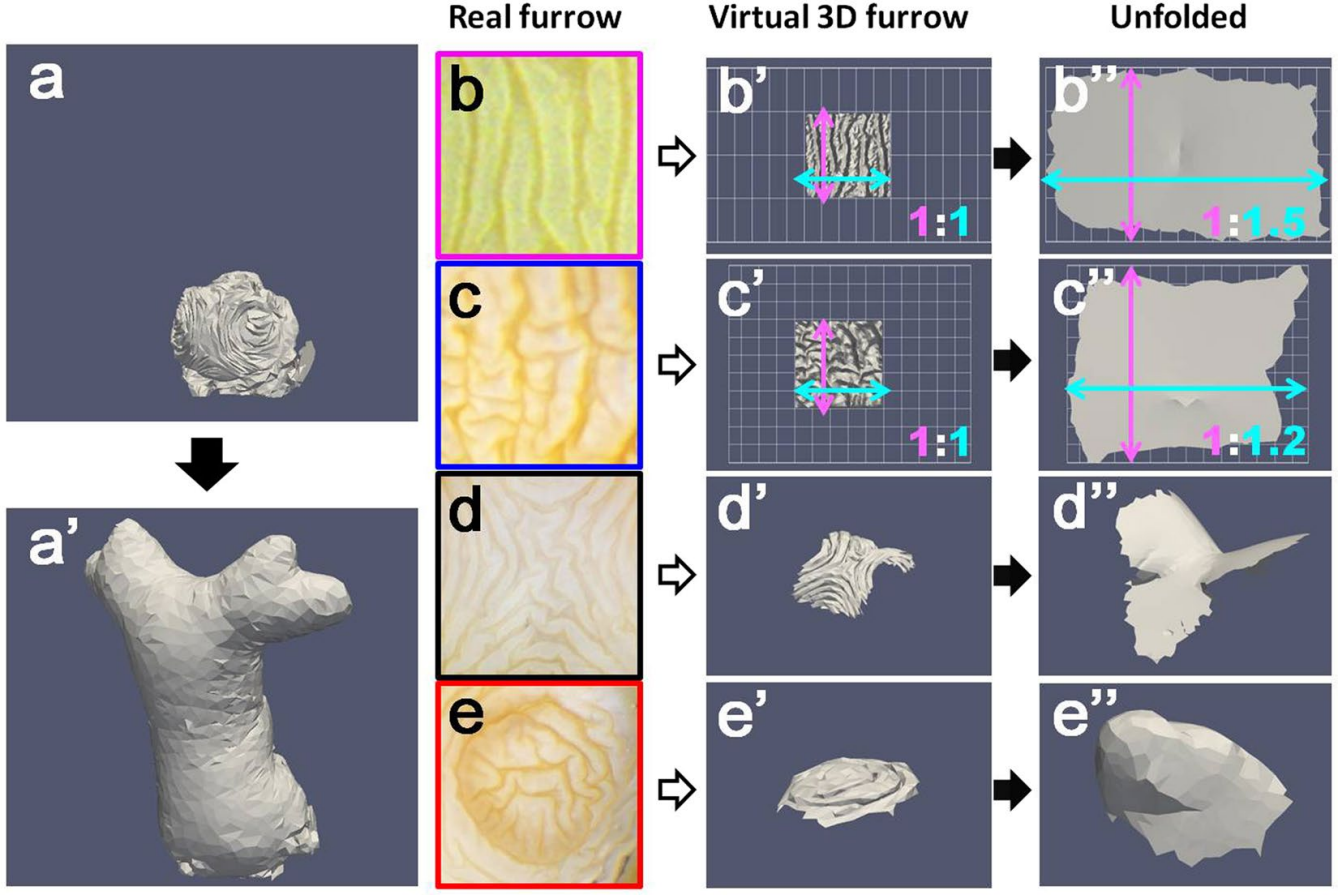
Computer simulation of horn primordia unfolding. (**a**) Virtual horn primordia. (a’) Unfolded virtual horn primordia. The characteristic 3D shape of the pupal horn with the bifurcated distal tip and the long column shaped stalk is correctly simulated by the algorithm. (**b**–**e**) Various furrow patterns observed *in vivo* (each colored window corresponds to the colored windows in Fig. 2). (b’–e’) Virtual 3D furrow patterns representing *in vivo* patterns. (b”–e”) Computer simulation of 3D furrow pattern unfolding. Note that the shape of an unfolded structure depends largely on its initial furrow pattern.

Using serial block-face imaging, we recorded the 3D folding pattern of the horn primordia *in vivo* as a 3D volume dataset (Fig. S1). To that end, fresh dissected tissue was embedded in a matrix for cryostat sectioning, whereupon the surface of the compound block (*not* a sliced tissue layer) was captured using a high resolution digital camera. This procedure ensures that any chemical damage (e.g. tissue shrinking during fixation) or physical damage (e.g. mechanical disruption of thin sliced tissues during sectioning) to the horn primordia tissue is kept at an absolute minimum. From the sequential 2D sections, a 3D mesh structure was constructed (Fig. S1). In our computer algorithm, the folded cuticle sheet of the virtual horn primordia is represented by multiple small triangular plates. To minimize the effects of cuticle elasticity, the size of each triangular plate is set as almost fixed (only a minimum size change is allowed for unfolding). On the other hand, the angles of adjacent triangles can be altered by subjecting each given point to mild tension. During computer simulations, the 3D structure of the horn primordia at the prepupal stage was set as the initial condition. Then virtual pressure from the inside was applied to all points in the mesh, thereby imitating the *in vivo* unfolding of the furrows during pupation (details of the simulation and the code of the simulator is available in the supplementary information file). As a result, the virtual horn primordia was successfully transformed into a virtual pupal horn with a 3D shape indistinguishable from its *in vivo* counterpart (Fig. 4a). Thus, we conclude that unfolding the furrow plays the major role in primordia to final horn transformation rather than local extension or compression of the cuticle sheet.

It is difficult to understand intuitively how complex furrows at the horn primordia surface are integrated in a coordinated developmental program that ultimately generates a beetle horn. Nevertheless, the impact of local furrow unfolding on the shape of an epithelial cell sheet should be predictable. Based upon *in vivo* horn primordia data (Fig. 4b–e), we calculated the 3D shape changes induced by the unfolding of some of the characteristic furrow patterns observed (Fig. 4b”–e”). For example, unfolding of simple parallel furrows results in widening of the 2D epithelial sheet in one direction (Fig. 4b’,b”), whereas unfolding of wandering (zigzag) furrows broadens the 2D sheet in every direction (Fig. 4c’,c”). On the other hand, unfolding of a furrow pattern as depicted in Fig. 4d, a region where two concentric rings meet, results in a horseback-like 3D shape (Fig. 4d”). The pattern shown in Fig. 4e corresponds to one of the tips of the horn, which adopts a dome-like structure when extended (Fig. 4e”). Consequently, generating the complex 3D shape of a beetle horn may be as simple as arranging furrows in a highly specific pattern on the horn primordia, which has originally a simple dome shape underpinning the larval head capsule.

### Furrows are formed by uneven growth of the epithelial cell sheet via passive buckling and/or sequential invagination

Next, we determined how the furrows of the horn primordia are generated. There are two possible mechanisms controlling the formation of furrows in a growing cell sheet. The first mechanism involves an uniform expansion of the cell sheet, causing buckling of the sheet as a result of the available space becoming restricted. In this case, furrows are made passively, and unfolding of the furrow simply flattens the cell sheet again. The second mechanism involves local growth of the cell sheet, thereby causing invagination and/or local buckling of the tissue. In this case, unfolding of the furrow can induce specific 3D structures as shown in Fig. 4a. Although it is already known that a horn primordia develops during the prepupal stage and that by the end of this period they have many furrows^15^, the details of furrow formation have never been investigated.

**Figure 5:**
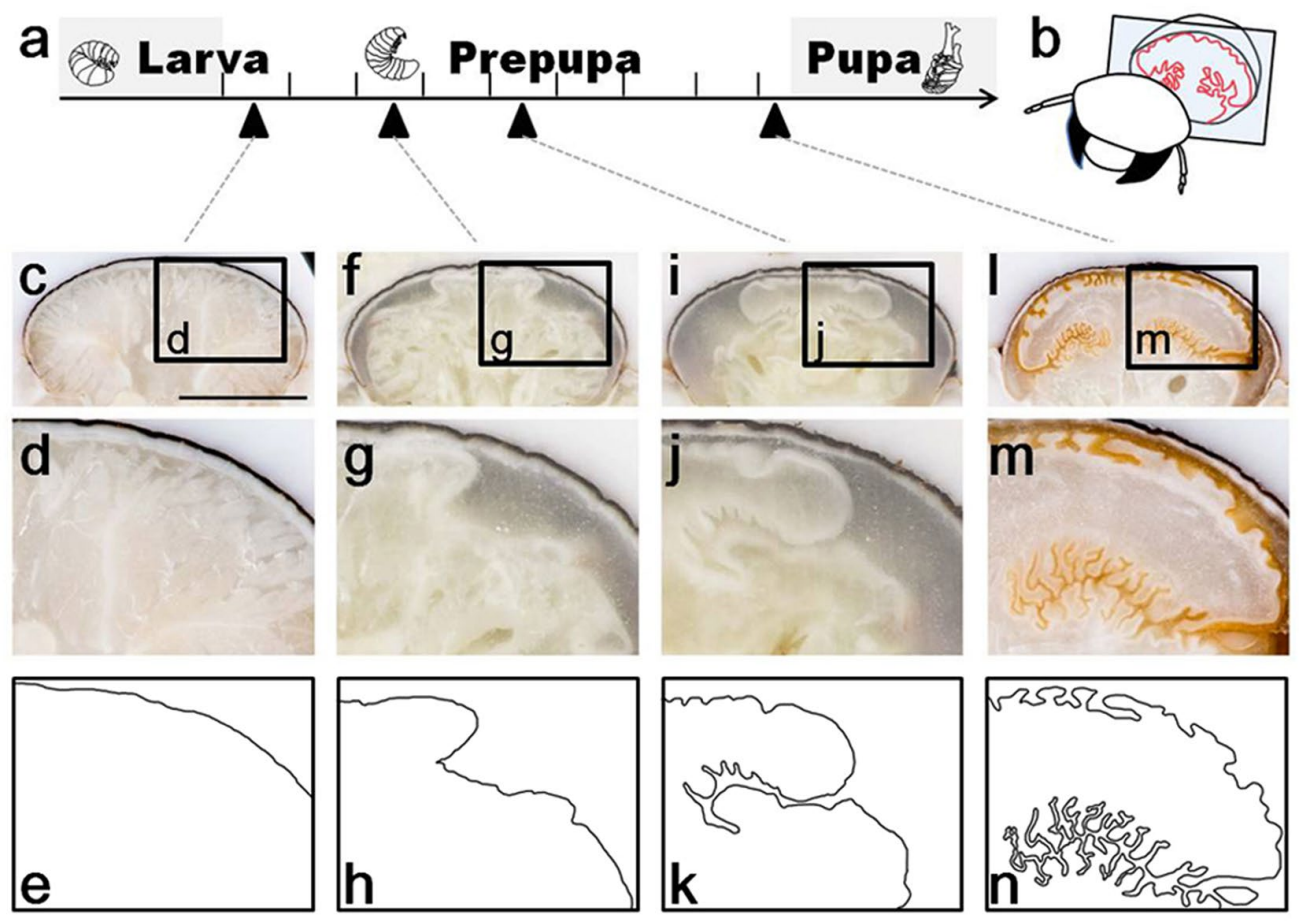
Furrows are formed by uneven growth of the horn primordia epithelial cell sheet (**a**). Developmental time window of the larval-pupal transformation in *Trypoxylus dichotomus*. During approximately eight days of the prepupal period, the horn primordia develops. (**b**) Schematic diagram of a frontal section of the horn primordia shown in (**c**,**f**,**i** and **l)**. (**c**,**f**,**i**,**l**) Frontal section of the whole prepupal head (black bar represents 5 mm). (**d**,**g**,**j**,**m**) Magnified image of the windows in (**c**,**f**,**i** and **l**), respectively. (**e**,**h**,**k**,**n**) Horn primordia surfaces are manually traced from (**d**,**g**,**j** and **m**), respectively. Note that the major furrow, which starts to be formed at prepupal day 3 (**f**–**h**), grows deeper and is branched (**i**–**k**).

Figure 5 shows frontal sections of the developing horn primordia during the prepupal stage. At the beginning of the prepupal stage, the 2D epithelial sheet does not yet adopt the characteristic horn-like structure, but simply covers the inner side of the larval cuticle (Fig. 5c,d). A few days later, invagination of the cell sheet begins to make a major furrow that separates the cap from the stalk region (Fig. 5f–h). At a later prepupal stage, many additional furrows branch off from the major furrow, thereby forming a complex nested folding structure. Also, entire cap region grows to be wider (Fig. 5i–k). Immediately before pupation, the furrow pattern adopts a highly complex structure. At this stage, the surface of the primordia has become covered with secreted pupal cuticle of a brownish color, indicating that the primordia has become less elastic (Fig. 5l,m). Apparently the growth speed of the primordia cells differs in a position dependent manner. This sequence of horn primordia development suggests that furrows are not made by passive buckling due to uniform expansion of the cell sheet, but are formed via region-specific growth and resulting in local buckling and/or active invagination.

## Conclusion

In this study, we show that the 3D shape of the horn of the Japanese rhinoceros beetle, *Trypoxylus dichotomus*, can be explained by the coordinated unfolding of furrows that are present at the surface of the horn primordia. Furrows in the exoskeleton just before the larval/pupal transition are widely observed among arthropods. During each molt the furrows are unfolded, whereupon the 3D shape becomes larger and sometimes more complex. Therefore, the furrows are likely to be involved in arthropod morphogenesis. However, the importance of this phenomenon has been largely overlooked, possibly due to lack of a suitably simple model system. Formation of the 3D shape in most animals involves the summation of many different cellular events, such as cell shape changes, proliferation and cell migration. As it is difficult to assess the contribution of each event and its integration into a coordinated developmental program that ultimately defines 3D structure, the study of 3D biological shape formation and unraveling its fundamental principles is hampered. However, in the case of beetle horn formation, we only need to focus on two events, which occur at different time points during development: formation of the furrows within the 2D epithelial sheet and their subsequent unfolding in 3D space. To generate the correct furrow pattern, morphogenetic mechanisms are needed that determine the direction, depth and shape of each furrow within the 2D epithelial sheet, allowing the formation of a pupal horn when the horn primordia is unfolded at a later stage. Because such processes can be considered as pattern formation in a 2D sheet, elucidating the mechanisms involved should be possible by molecular biological techniques. For example, various horn shapes are actually inducible by specific gene knockdown via RNAi in this species^16,19,20^. Exploring if/how altered expression of these and other genes affects local furrow patterns within developing horn primordia may dramatically improve our ability to link specific developmental pathways with final horn shape.

There are accumulated genetic and molecular information related to the patterning and the growth of beetle horn primordia^17–22^. However, the contribution of such data to the 3D shapes has remained obscure because of the lack of the clue to directly combine the 2D pattern in the primordia to the horn 3D shape. We expect that our computer simulation system may provide a clue to assess the contribution of such data to the horn 3D morphogenesis.

Moreover, the coordinated unfolding of the furrows during beetle horn formation is a process entirely driven by physical forces, enabling us to deduce the final 3D shape based upon an initial pattern of furrows (Fig. 4). On the other hand, the reverse question, namely how to design a furrow pattern that, when eventually unfolded, produces an arbitrarily chosen 3D shape, is of considerable mathematical interest. In the case of the beetle horn primordia, the 3D shape is made by the coordinated unfolding of given set of furrows. Taken together, we postulate that beetle horn formation is an ideal system to investigate how the 3D shape of animals is formed.

## Materials and Methods

### Insects

Larvae of *Trypoxylus dichotomus* were commercially purchased. Third instar larvae were individually bottled with rotted wood flake (Hercules mat, Mushiya.com, Osaka, Japan), kept at 10–15 °C (to suspend their development) and moved to 25 °C when needed. Bottles were checked daily and formation of the pupal case was recorded. We defined the beginning of the prepupal stage as the time point where construction of the pupal case was finished. Prepupae were used for horn primordia time-course observation via direct dissection, cryostat sections and Micro-CT scan.

### Dissection of developing horn primordia

Larvae were anesthetized on ice before dissection. Dissected horn primordia were observed under a binocular microscope (SZ61-TRC, Olympus, Tokyo, Japan) and pictures captured by a digital camera (EOS DIGITAL, Canon, Tokyo, Japan).

### Sectioning of developing horn primordia

We observed horn primordia development via sectioning by serial block-face imaging^23^. This method protects horn primordia structures from any chemical and physical damages (Fig. S1). Dissected larval heads were mounted in OCT compound (Sakura Finetek, Tokyo, Japan). Frozen blocks were sectioned with cryostat (Leica CM3050S, Leica Microsystems K.K., Tokyo, Japan) and pictures of the block surface were automatically taken by a digital camera (Nikon D810, Tokyo, Japan) after every section (20 µm). The chamber temperature was set at −15 °C.

### Micro-CT scan of horn primordia

Sample preparation was performed as modified from previous studies^24,25^. Prepupal heads were fixed overnight by Bouin solution and transferred to 70% ethanol. Then samples were dehydrated by a series of ethanol up to 100%. For staining, a 3% iodine/ethanol solution was used. Samples were dried and scanned with a micro-CT scanner (inspeXio SMX100CT, Shimazu, Kyoto, Japan).

### Histochemistry

Day 8 prepupae were chemically fixed with formalin and horn primordia were dissected in PBS. After replacement into a 50% sucrose solution, horn primordia were embedded in OCT compound (Sakura Finetek, Tokyo, Japan) and frozen. A cryostat section (4 µm thickness) was stained 1:1,000 with Hoechst 33342 Reference standard (A22283, Invitrogen) to PBS dilution.

### Artificial horn primordia extension

Dissected horn primordia were fixed with formalin for a week, after which the horn primordia was cut at the base of the stalk. Internal denatured tissues were removed and a plastic tube was attached to the horn primordia by a silicone string.

### Construction of 3D image from serial section images

Captured serial block-face images (N = 273) were digitally processed and the borders of the newly formed pupal primordia were manually traced by Photoshop software (Adobe Systems, San Jose, CA, USA). The surface mesh of the horn primordia was reconstructed using 3D slicer software (^26^, www.slicer.org). The entire shape of the horn primordia is represented by the surface mesh composed of facets, edges, and vertices. Using Meshlab software (^27^, http://meshlab.sourceforge.net/), the quadric edge collapse decimation filter was applied to reduce the number of vertices. A mesh of a sheet with a local furrow pattern was prepared by cropping the entire mesh.

### Energy minimization for simulating the unfolding of the horn primordia

In order to simulate the unfolding of the horn primordia, energy minimization is performed with respect to the vertex position using the steepest decent method^28^:

1 $${\rm{\gamma }}\frac{d{{\boldsymbol{r}}}_{{\boldsymbol{i}}}}{dt}=-\frac{\partial U}{\partial {{\boldsymbol{r}}}_{{\boldsymbol{i}}}}$$

where γ is a friction coefficient and ***r***_*i*_ is the position vector of vertex *i*. The total energy function *U* is the sum of elastic energies stored by changes in polygon volume, facet area, edge length, and inter-facet angle from their stress-free state (initial state).

### Unfolding of the entire horn primordia

To simulate the unfolding of the entire horn primordia by volume expansion under area constraint of the primordia, we employ higher elastic constants with respect to facet area and edge length changes, and increase the internal pressure of the polygon by increasing the polygon volume at stress-free state *V*_0_, with time *t* as follows,

2 $${V}_{0}=3{V}_{init}\,(1-\frac{2}{3}{e}^{-t})$$

where *V*_*init*_ is the initial polygon volume.

### Unfolding of the sheet with a local furrow pattern

To perform unfolding of the sheet with a local furrow pattern, we set the stress-free state of inter-facet angle as *π* [rad], and perform energy minimization. Other model parameters are available in Supplementary Information.

### Mesh for calculation of the magnification ratio

Images of furrow patterns were obtained by stereomicroscope and processed by Fiji^29^. They were cropped and resized into 100 × 100 pixels. After resizing, they were converted to 8-bit grayscale. Meshes were constructed from the 8-bit grayscale text images. The position of each vertex was determined by the location and tone of the corresponding pixel. The post mesh process was performed using Meshlab, in which a HC Laplacian smooth filter was applied twice and a quadric edge collapse decimation filter was applied three times to smoothen the surface and reduce the number of vertices, respectively.

## Electronic supplementary material

Supplementary information

Supplementary Movie 1

Supplementary Movie 2

## References

[1] Chapman, R. F. The insects: structure and function. (Cambridge university press, 1998).

[2] Snodgrass, R. E. Principles of insect morphology. (Cornell University Press, 1993).

[3] Bernays E (1972). Changes in the first instar cuticle of Schistocerca gregaria before and associated with hatching. J. Insect Physiol..

[4] Fristrom, D., Fristrom, J. W. The metamorphic development of the adult epidermis. *The development of* Drosophila *melanogaster* (eds Bate, M., Martinez-Arias, A.) vol 2, 843–897 (Cold Spring Harbor Lab. Press, 1993).

[5] Morata G (2001). How *Drosophila* appendages develop. Nat. rev. Mol. cell biol..

[6] Johnston LA, Gallant P (2002). Control of growth and organ size in *Drosophila*. BioEssays..

[7] Vincent JP, Fletcher AG, Baena-Lopez LA (2013). Mechanisms and mechanics of cell competition in epithelia. Nat. rev. Mol. cell biol..

[8] Wartlick O, González-Gaitán M (2011). The missing link: implementation of morphogenetic growth control on the cellular and molecular level. Curr. Opin. Genet. Dev..

[9] Romanova‐Michaelides M, Aguilar‐Hidalgo D, Jülicher F, Gonzalez‐Gaitan M (2015). The wing and the eye: a parsimonious theory for scaling and growth control?. WIREs Dev. Biol..

[10] Fristrom D, Fristrom JW (1975). The mechanism of evagination of imaginal discs of *Drosophila melanogaster*: I. General considerations. Dev. Biol..

[11] Cohen, S. M. Imaginal disc development. *The development of* Drosophila *melanogaster* (eds Bate, M., Martinez-Arias, A.) vol 2, 747–841 (Cold Spring Harbor Lab. Press, 1993).

[12] Fristrom D, Chihara C (1978). The mechanism of evagination of imaginal discs of *Drosophila melanogaster*: V. Evagination of disc fragments. Dev. Biol..

[13] Fristrom D (1988). The cellular basis of epithelial morphogenesis. A review. Tissue and Cell..

[14] Condic ML, Fristrom D, Fristrom JW (1991). Apical cell shape changes during *Drosophila* imaginal leg disc elongation: a novel morphogenetic mechanism. Development..

[15] Emlen DJ, Lavine LC, Ewen-Campen B (2007). On the origin and evolutionary diversification of beetle horns. Proc. Nat. Acad. Sci. USA.

[16] Emlen DJ, Warren IA, Johns A, Dworkin I, Lavine LC (2012). A mechanism of extreme growth and reliable signaling in sexually selected ornaments and weapons. Science..

[17] Moczek AP, Nagy LM (2005). Diverse developmental mechanisms contribute to different levels of diversity in horned beetles. Evol. Dev..

[18] Moczek AP, Andrews J, Kijimoto T, Yerushalmi Y, Rose DJ (2007). Emerging model systems in evo‐devo: horned beetles and the origins of diversity. Evol. Dev..

[19] Ito Y (2013). The role of *doublesex* in the evolution of exaggerated horns in the Japanese rhinoceros beetle. EMBO rep..

[20] Gotoh H (2015). The Fat/Hippo signaling pathway links within‐disc morphogen patterning to whole‐animal signals during phenotypically plastic growth in insects. Dev. Dyna..

[21] Kijimoto T, Pespeni M, Beckers O, Moczek AP (2013). Beetle horns and horned beetles: emerging models in developmental evolution and ecology. WIREs Dev. Biol..

[22] Ledón-Rettig, C. C., Zattara, E. E., Moczek, A. P. Asymmetric interactions between doublesex and tissue-and sex-specific target genes mediate sexual dimorphism in beetles. Nat. Comm. 8 (2017). 10.1038/ncomms14593 PMC5333360 28239147

[23] Tajika Y (2017). A novel imaging method for correlating 2D light microscopic data and 3D volume data based on block-face imaging. Sci. Rep..

[24] Metscher BD (2009). MicroCT for comparative morphology: simple staining methods allow high-contrast 3D imaging of diverse non-mineralized animal tissues. BMC Physiol..

[25] Metscher BD (2009). MicroCT for developmental biology: A versatile tool for high‐contrast 3D imaging at histological resolutions. Dev. Dyna..

[26] Fedorov A (2012). 3D Slicer as an image computing platform for the quantitative imaging network. Magnetic Resonance Imaging..

[27] Cignoni, P. *et al*. Meshlab: an open-source mesh processing tool. In Eurographics Italian Chapter Conference Vol. 2008: 129–136 (2008).

[28] Bartholomew-Biggs, M. C. Nonlinear optimization with engineering applications. Vol. 19 (Springer Science & Business media, 2008).

[29] Schindelin J (2012). Fiji: an open-source platform for biological-image analysis. Nat. methods..

