# Supplementary material for: Complex furrows in a 2D epithelial sheet code the 3D structure of a beetle horn
Source: Sci Rep. 2017 Oct 24;7:13939. doi: 10.1038/s41598-017-14170-w (PMC5655322; doi:10.1038/s41598-017-14170-w)

**Supplementary information**

**Complex furrows in a 2D epithelial sheet code the 3D structure of a beetle horn**

Keisuke Matsuda^1^, Hiroki Gotoh^2*^, Yuki Tajika^3^, Takamichi Sushida^4^, Hitoshi Aonuma^4^, Teruyuki Niimi ^5^, Masakazu Akiyama^4^, Yasuhiro Inoue^6^, Shigeru Kondo^1^

Matsuda and Gotoh contributed equally.

1. Graduate School of Frontier Bioscience, Osaka University, Suita, Osaka, 565-0871, Japan

2. Graduate School of Bioagricultural Sciences, Nagoya University, Nagoya, Aichi, 464-8601, Japan

3. Graduate School of Medicine, Gunma University, Maebashi, Gunma, 371-8511, Japan

4. Research Institute for Electronic Science, Hokkaido University, Sapporo, Hokkaido, 060-0812, Japan

5. Division of Evolutionary Developmental Biology, National institute for basic biology, Okazaki, Aichi, 444-8585, Japan

6. Institute for Frontier Life and Medical Sciences, Kyoto University, Kyoto, Kyoto, 606-8507, Japan

* Corresponding author. *Hiroki Gotoh*, *Graduate School of Bioagricultural Sciences, Nagoya University, Chikusa, Nagoya 464-8601, Japan*

E-mail address: h-r-goto@ees.hokudai.ac.jp (H. Gotoh)

Supplementary Figure S1: Schematic diagram of a virtual 3D horn primordia via binarized images, constructed from serial block-face images

Supplementary Table and text for simulation

Supplementary movie 1: CT-Scanned fully developed horn primordia

Supplementary movie 2: Blowing up of the horn primordia


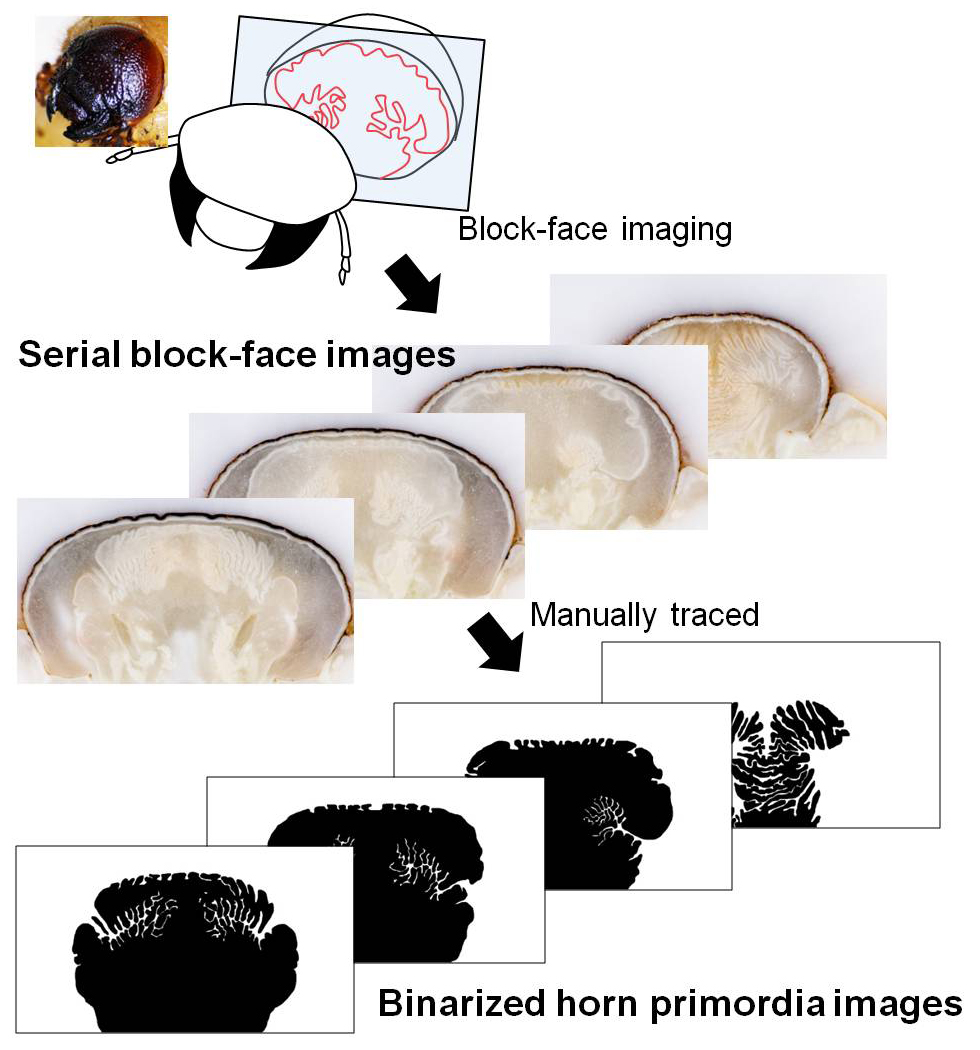


**Figure S1.**

Schematic diagram of a virtual 3D horn primordia via binarized images, constructed from serial block-face images. The larval head was frontally sectioned using a cryostat and series of serial block-face images were obtained. The horn primordia was traced manually in all serial block-face images in order to convert them to binarized images. By using series of binarized images, virtual 3D primordia was constructed (Fig. 4a) and was able to be extended (Fig. 4a’).

This serial block-face imaging method protect horn primordia structures from any chemical damage (e.g. tissue shrinking during fixation) or physical damage (e.g. mechanical disruption of thin sliced tissues during sectioning). Thus, we employed this methods instead of Micro-CT scanning in which horn primordia structures were somewhat changed during sample preparation (chemical fixation and freeze drying).

**Supplementary Table and text for simulation**

Total energy


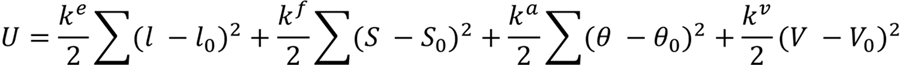


Model parameters: Unfolding of the entire imaginal primordia

| Symbol | Value | Descriptions |
| --- | --- | --- |
| k^e^ | 1e+5 | Constant of edge elasticity |
| k^f^ | 10.0 | Constant of facet elasticity |
| k^a^ | 0.1 | Constant of inter-facet angle elasticity |
| k^v^ | 1e-4 | Constant of volume elasticity |
| *γ* | 5.0 | Friction coefficient |

Model parameters: Unfolding of the sheet with the local furrow pattern

| Symbol | Value | Descriptions |
| --- | --- | --- |
| k^e^ | 1e+2 | Constant of edge elasticity |
| k^f^ | 40.0 | Constant of facet elasticity |
| k^a^ | 1e+4 | Constant of inter-facet angle elasticity |
| k^v^ | 0.0 | Constant of volume elasticity |
| *γ* | 5.0 | Friction coefficient |

Supplementary movie 1: CT-Scanned fully developed horn primordia


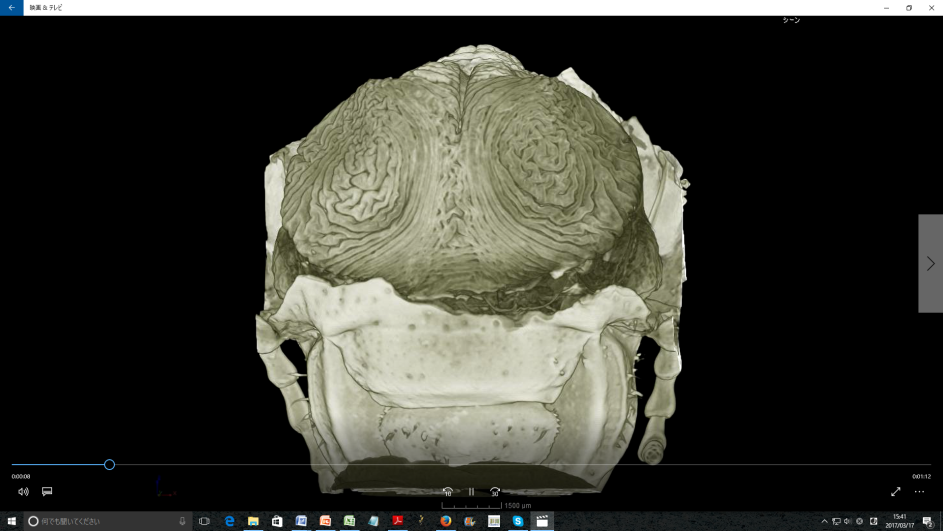


Supplementary movie 2: Blowing up of the horn primordia


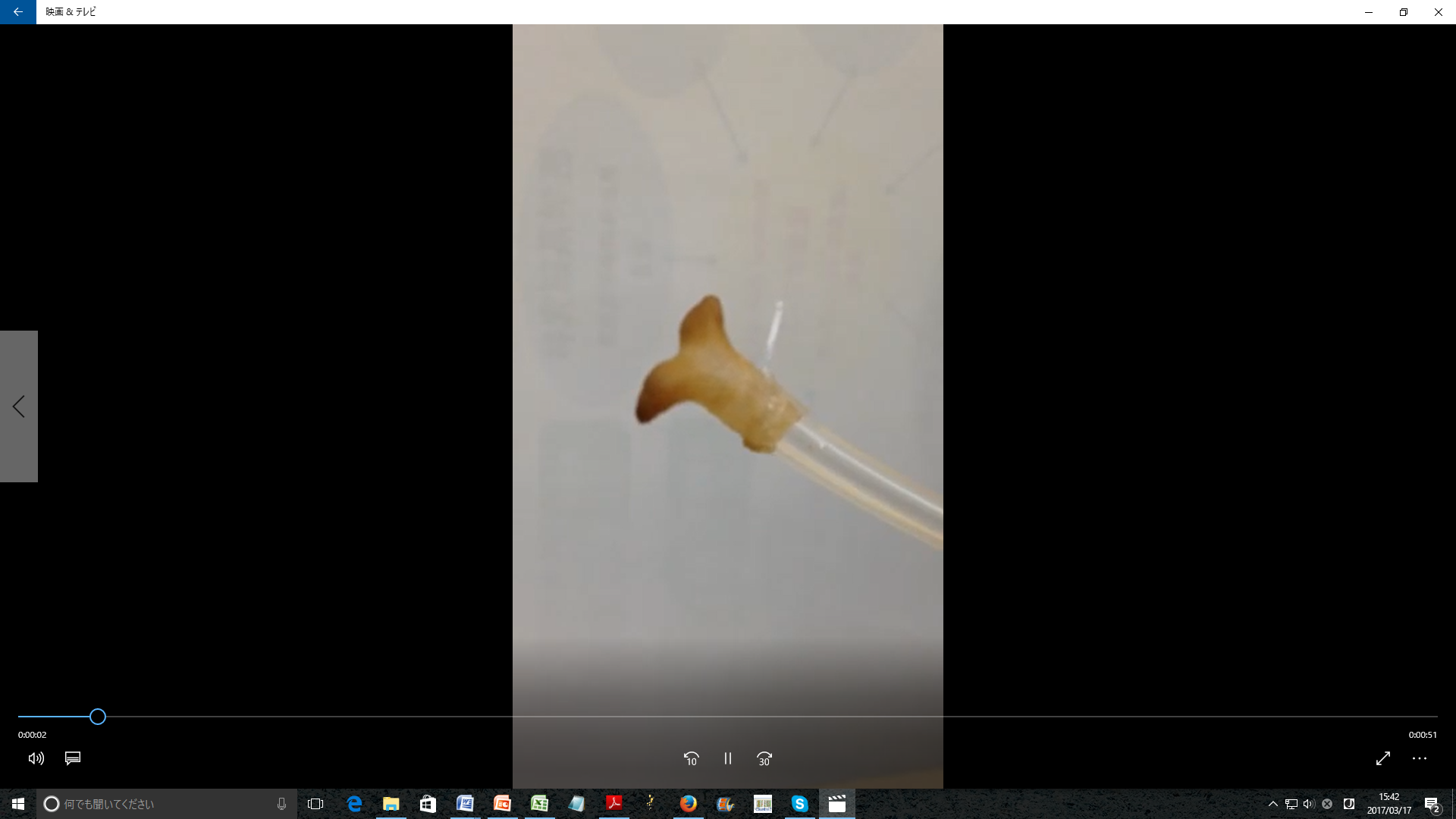

Supplement: Supplementary file 1 — Supplementary information [file 41598_2017_14170_MOESM1_ESM.doc]
